# The Salivary Microbiota, Cytokines, and Metabolome in Patients with Ankylosing Spondylitis Are Altered and More Proinflammatory than Those in Healthy Controls

**DOI:** 10.1128/mSystems.01173-20

**Published:** 2021-06-22

**Authors:** Longxian Lv, Huiyong Jiang, Ren Yan, Danyi Xu, Kaicen Wang, Qiangqiang Wang, Xiaoxiao Chen, Lanjuan Li

**Affiliations:** a State Key Laboratory for Diagnosis and Treatment of Infectious Diseases, National Clinical Research Center for Infectious Diseases, Collaborative Innovation Center for Diagnosis and Treatment of Infectious Diseases, The First Affiliated Hospital, College of Medicine, Zhejiang University, Hangzhou, China; b Department of Rheumatology, The First Affiliated Hospital, College of Medicine, Zhejiang University, Hangzhou, China; University of Pennsylvania

**Keywords:** ankylosing spondylitis, salivary microbiota, immunity, cytokines, metabolome

## Abstract

The pathogenesis of ankylosing spondylitis (AS) remains unclear but appears to be associated with heredity and the environment. The mouth links the external environment to the gut and lungs. In the present study, compared to that observed in healthy controls (HCs), AS saliva was depleted of Bacilli such as Streptococcus, enriched with Clostridia such as *Veillonellaceae*, and enriched with opportunistic pathogens from *Proteobacteria* such as Brucella spp. and Campylobacter concisus. AS saliva was enriched with 16 cytokines related to inflammation, such as soluble IL-6 receptor α (sIL-6Rα), interleukin 2 (IL-2), IL-10, IL-11, IL-12p40, IL-12p70, IL-20, IL-26, IL-27, IL-28A, IL-29, alpha 2 interferon (IFN-α2), IFN-β, and matrix metalloproteinase 3 (MMP-3). AS saliva was also enriched with hazardous compounds, such as cadaverine and putrescine. AS-altered salivary bacteria, compounds, and cytokines are closely linked with disease indicators. Oral cleaning reduced the levels of proinflammatory cytokines and hazardous compounds in AS saliva compared with HC saliva. AS saliva induced the production of more proinflammatory cytokines, such as IL-12p70 and IL-8, by THP-1 monocyte-derived macrophages, than did HC saliva. The results highlight the importance of salivary microbes, cytokines, and compounds in the development and treatment of AS and provide new ideas for the pathogenesis and treatment of AS.

**IMPORTANCE** Ankylosing spondylitis (AS) affects as much as 0.32% of the population in some districts and causes work disability in one-third of these patients. Microbes are considered to play important roles in AS pathogenesis, and the mouth links the environment to the lungs and the gut. Our results showed that opportunistic pathogens such as Brucella and Campylobacter are enriched in the saliva of AS patients with ankylosing spondylitis. In addition, proinflammatory cytokines and hazardous materials such as putrescine were also enriched in the saliva of AS patients.[AQ1 sentence edit] Interestingly, the opportunistic pathogens and hazardous materials detected in the saliva of AS patients were associated with disease indexes. The saliva of AS patients was shown to induce immune cells to secrete proinflammatory cytokines *in vitro*. Reducing the levels of salivary microbes can significantly reduce the hazardous materials present in the saliva of AS patients. Our results provide a new perspective on the potential role of salivary microbes, cytokines, and hazardous compounds in the pathogenesis and treatment of AS.

## INTRODUCTION

Ankylosing spondylitis (AS) is a male-predominant chronic inflammatory spondyloarthropathy that primarily damages the sacroiliac joints and/or the spine and sometimes invades the peripheral joints and other organs (1, 2). The prevalence of AS ranges from as high as 0.07% in Africa to 0.32% in North America, but its incidence is increasing (2). Furthermore, approximately one-third of AS patients report disease-related work disability due to its current unclear pathogenesis and incurable characteristics (3).

The pathogenesis of many autoimmune diseases, including AS, is associated with heredity and the environment and is a process in which microbes are considered to play important roles (4, 5). On one hand, associations of AS with microbes have frequently been observed. Childhood respiratory tract infections are positively associated with the prevalence of AS (1). The level of gut Klebsiella pneumoniae is also positively associated with the occurrence and activity of AS. Serum IgA antibodies against Klebsiella pneumoniae have been detected in AS patients (6). A metagenomic study reported that Prevotella melaninogenica is enriched and *Bacteroides* spp. are depleted in the feces of AS patients versus healthy controls (HCs) (7). Furthermore, in AS animal models, *HLA-B27* transgenic rats do not develop chronic enteritis, peripheral arthritis, or caudal spondylitis in a sterile environment as they do in a normal environment (8). On the other hand, several potential mechanisms by which microbes affect AS have been reported. For instance, microbes can produce antigens similar in sequence or structure to those of the host, which may trigger AS through molecular mimicry. For example, homologies have been observed between Klebsiella pneumoniae nitrogenase (residues 188 to 193) and HLA-B27.1 antigen (residues 72 to 77) (8). In addition, microbes may affect the IL-23/IL-17 immune axis, which is important for the immunopathogenesis of AS. For example, Chlamydia trachomatis-induced IL-23 activation is associated with reactive arthritis (9). Moreover, local Th17 responses can be stimulated by Salmonella enteritidis in animal reactive arthritis models (10).

The mouth links the external environment to the lung and gut, which is a crucial factor in health and diseases. The oral microbiota is the second-largest human microbiota. Nearly 1,000 bacterial species colonize the oral cavity and are distributed in saliva, the buccal mucosa, the palatal mucosa, the tongue dorsum, dental plaque, and other places, with the bacteria present in saliva displaying the highest abundance and diversity. The oral microbiota has important functions, such as acting as a barrier and regulating immunity and metabolism. Moreover, the oral cavity bridges the gap between exogenous substances and the internal environment. Approximately 10^11^ to 10^12^ bacteria enter the digestive tract in approximately 1.5 liters of saliva per day for adults (11, 12). Dental bacteria penetrate and thus potentially affect the integrity of the oral mucosa. Consequently, the oral microbiota affects both oral and extraoral health and disease. Numerous reports have shown that some members of the oral microbiota may be involved in many diseases, such as autoimmune diseases and colorectal cancer, in addition to being important causes of periodontitis, dental caries, and other oral diseases (13–17).

Patients with AS show a higher prevalence of periodontitis and poor oral hygiene than healthy controls (18), as well as a worse oral health-related quality of life (19). Furthermore, AS populations are reported to have an increased risk of reporting oral ulcers (20). The goal of the present study was to determine the alterations in the microbiota, immunity, and metabolism in the saliva of AS patients and to elucidate the role of these alterations in the occurrence and development of AS.

## RESULTS

### Demographic and clinical characteristics.

Seventy-eight participants were enrolled, including 37 patients with AS and 41 sex- and age-matched HCs. All patients were carefully screened and did not suffer from diseases or health problems other than AS (Table 1). In the AS group, 6/37 (16.2%) patients did not take any drugs; 8/37 (21.6%) patients were receiving nonsteroidal anti-inflammatory drugs (NSAIDs); 6/37 (16.2%) patients were receiving sulfasalazine; 10/37 (27.0%) patients were receiving both NSAIDs and sulfasalazine; and 7/37 (18.9.2%) patients were receiving tumor necrosis factor α (TNF-α) inhibitors. The median Bath ankylosing spondylitis activity disease activity index (BASDAI) and AS disease activity score (ASDAS) scores were 3.05 and 2.28, respectively. Compared to those observed in HCs, the erythrocyte sedimentation rate (ESR), C-reactive proteins (CRP), granulocytes, neutrophils, platelet distribution width, total protein, globin, alkaline phosphatase, and γ-glutamyltransferase were increased, whereas the lymphocytes, eosinophils, mean erythrocyte hemoglobin concentration, mean platelet volume, direct bilirubin, indirect bilirubin, and creatinine levels were decreased in AS patients.

**TABLE 1:** Demographic and clinical characteristics of the study population

Variable^*a*^	AS^*a*^	HC^*a*^	*P* value
Total *n* (no. female)	37 (13)	41 (14)	-
Age (yrs ± SD)	33.86 ± 1.85	33.32 ± 1.71	8.28E−01
Time from symptom onset (months [IQR])	14.50 (3.00, 59.75)	-	-
Back pain score (IQR)	4.00 (2.00, 4.00)	-	-
Duration of morning stiffness (min [IQR])	14.50 (3.00, 59.75)	-	-
HLA-B27 positive (no. negative)	35 (2)	-	-
Family history (negative/positive)	35/2	-	-
BASDAI (IQR)	3.05 (2.13, 3.88)	-	-
ASDAS (IQR)	2.28 (1.52, 3.05)	-	-
Erythrocyte sedimentation rate (mm/h ± SD)	16.41 ± 2.66	0.82 ± 0.09	4.00E−06
C-reactive protein (mg/liter [IQR])	7.08 (1.79, 15.81)	2.00 (1.00, 2.00)	6.00E−03
Granulocyte (10^9^/liter ± SD)	4.85 ± 0.32	3.22 ± 0.27	1.08E−03
Neutrophil (% ± SD)	67.73 ± 1.10	55.91 ± 2.41	1.86E−04
Lymphocyte (% ± SD)	24.59 ± 0.99	35.15 ± 1.87	2.70E−06
Eosinophil (% ± SD)	0.80 (0.60, 1.60)	1.60 (1.15, 2.80)	1.20E−02
Mean erythrocyte hemoglobin concn (g/liter ± SD)	324.93 ± 3.32	335.94 ± 3.03	2.77E−02
Platelet distribution width (% [IQR])	15.80 (12.50, 16.20)	13.00 (11.75, 15.00)	2.70E−02
Mean platelet vol (fl [IQR])	10.50 (9.80, 10.80)	11.00 (10.35, 11.75)	2.00E−02
Total protein (g/liter ± SD)	77.23 ± 0.83	73.02 ± 0.59	1.00E−03
Globin (g/liter ± SD)	29.33 ± 0.77	24.27 ± 0.56	1.65E−05
Alkaline phosphatase (U/liter ± SD)	82.21 ± 3.66	64.00 ± 4.51	3.09E−03
Direct bilirubin (mg/dl [IQR])	3.00 (2.00, 3.00)	5.00 (4.00, 7.00)	1.37E−04
Indirect bilirubin (mg/dl [IQR])	5.00 (4.00, 7.75)	9.00 (7.50, 12.00)	1.04E−03
γ-Glutamyltransferase (U/liter [IQR])	19.50 (14.25, 29.5)	15.00 (11.50, 18.00)	3.30E−02
Creatinine (mg/dl ± SD)	68.24 ± 3.01	83.58 ± 3.43	3.86E−03

a IQR, interquartile range; SD, standard deviation; BASDAI, Bath ankylosing spondylitis activity disease activity index; ASDAS, AS disease activity score; AS, ankylosing spondylitis; HC, healthy control; -, not relevant.

### Salivary microbiota are different between AS patients and HCs.

**FIG 1:**
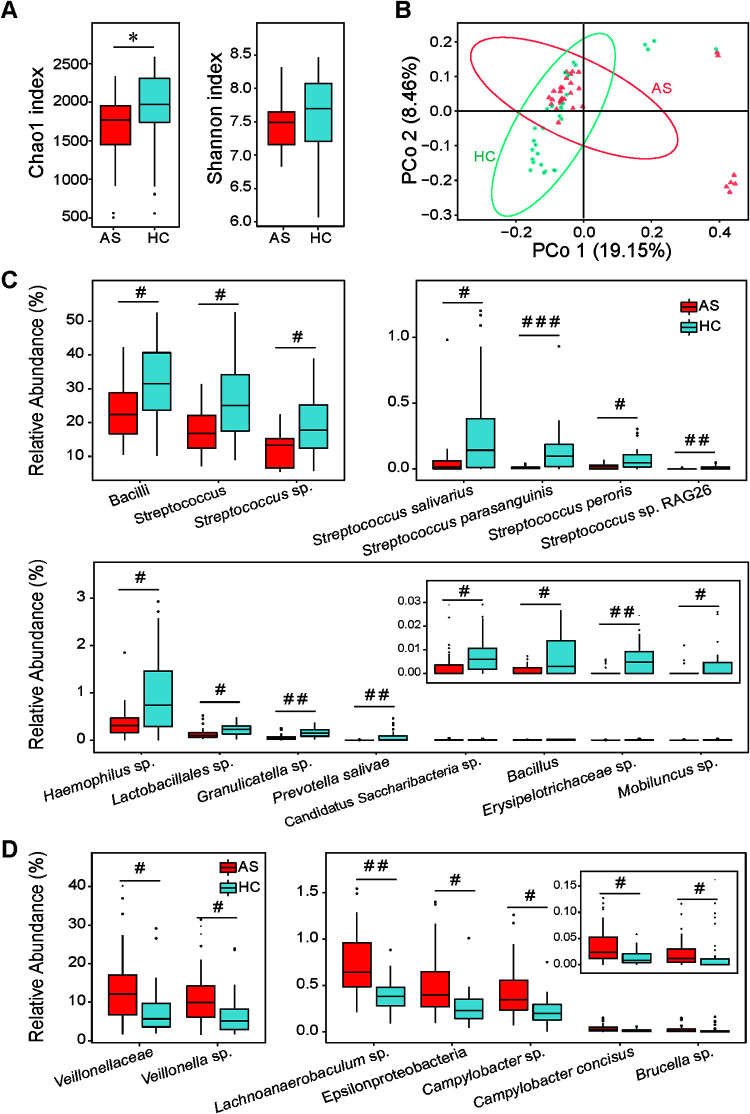
Altered microbiota diversity and composition in the saliva of patients with AS. (A) Chao1 index and Shannon index describing the alpha diversity of the salivary microbiota in AS patients and HC individuals. (B) β-Diversity of AS and HC salivary microbiota illustrated by PCoA built based on the unweighted UniFrac matrix. (C) Salivary bacterial taxa depleted in AS patients versus HCs. (D) Salivary bacterial taxa enriched in AS patients versus HCs. *, *P < *0.05; **, *P* < 0.01; ***, *P* < 0.001; #, q < 0.05; ##, q < 0.01; ###, q < 0.001.

To investigate the alterations in the salivary microbiota in AS patients, we first performed 16S rRNA gene metagenomic sequencing and obtained a mean of 38,230 reads for each sample. Bioinformatics analysis showed that the salivary microbiota of these participants mainly consisted of 13 bacterial phyla. In HCs, the top six phyla ranked by the average relative abundance were *Firmicutes* (mean relative abundance of 44.20%), *Proteobacteria* (20.56%), *Bacteroidetes* (18.39%), *Actinobacteria* (10.70%), *Fusobacteria* (4.23%), and *Candidatus Saccharibacteria* (0.92%). The Chao1 index of AS patients was significantly lower than that of HCs, indicating that AS patients had a reduced mean community richness. However, there was no significant difference in the Shannon index between the two groups, indicating that their species diversities were similar (Fig. 1A). Principal coordinates analysis (PCoA) showed that samples were clustered closely within the AS or HC groups but dispersed to a greater extent between groups (Fig. 1B). This finding illustrates that the difference between the two groups is greater than that within each group, and the two groups are dissimilar. The permutational multivariate analysis of variance (PERMANOVA) result (*P = *0.003) further demonstrated that the difference between the two groups was significant.

Compared to that observed in HC saliva, nearly 68% of all taxa altered in AS patient saliva belong to the phylum *Firmicutes*, which might be a result of the relative abundance of *Firmicutes* ranking first among all phyla. All altered taxa belonging to the class Bacilli appeared as depleted taxa in AS patients, among which 77% were members of the order *Lactobacillales*. These depleted taxa included the genus *Bacillus*, the unclassified species *Granulicatella* sp., two unclassified operational taxonomic units (OTUs) belonging to the order *Lactobacillales* or the family *Erysipelotrichaceae*, the genus Streptococcus, the species Streptococcus parasanguinis, Streptococcus peroris, Streptococcus salivarius, and Streptococcus sp., and the strain Streptococcus sp. RAG26 (Fig. 1C). The relative abundance of Streptococcus (AS versus HC, 17.85 ± 1.25 versus 26.65 ± 1.98) was the highest among all depleted taxa, and Streptococcus sp. (12.06 ± 0.91 versus 19.77 ± 1.68) contributed the most to this depletion. In addition, the family *Veillonellaceae* and the species *Veillonella* sp. and *Lachnoanaerobaculum* sp. belonging to the order *Clostridiales* were significantly enriched in AS saliva. Among them, *Veillonellaceae* (AS versus HC, 13.99 ± 1.72 versus 8.14 ± 1.21) was the most enriched, nearly 85% of which was contributed by *Veillonella* sp. (Fig. 1D).

Four taxa of the phylum *Proteobacteria* were enriched in AS saliva, while one was depleted. Most members belonging to the enriched taxa, such as the class *Epsilonproteobacteria* and the species Brucella sp., Campylobacter concisus, and Campylobacter sp., are opportunistic pathogens (Fig. 1D). In contrast, the species *Haemophilus* sp. was depleted. In addition, the mean relative abundance of the species Prevotella salivae belonging to the phylum *Bacteroidetes* was decreased approximately 190-fold. In addition, the unclassified species *Mobiluncus* sp., belonging to the phylum *Actinobacteria*, and an OTU belonging to the phylum *Candidatus Saccharibacteria* were also depleted in AS patients (Fig. 1C).

### Salivary cytokines are significantly enriched in AS patients versus HCs.

**FIG 2:**
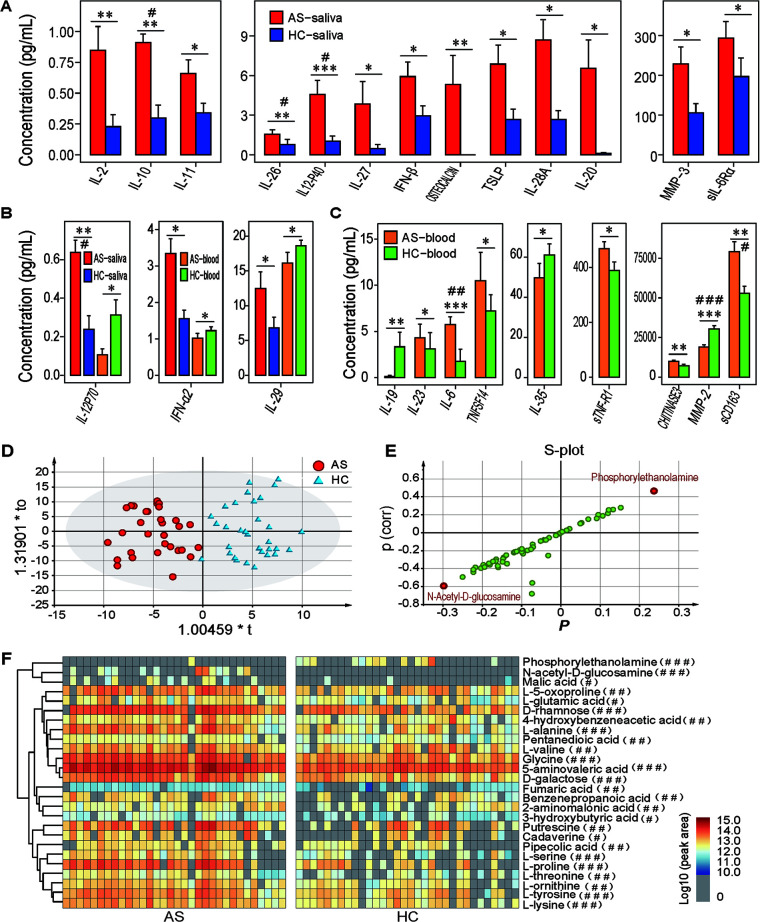
Altered salivary cytokines and metabolome profiles in patients with AS. (A) Cytokines enriched only in AS saliva but without differences in levels between AS and HC serum. (B) Cytokines enriched in AS saliva but depleted in AS serum in comparison to those detected in HC saliva or serum. (C) Cytokines depleted in AS serum but not AS saliva. (D) OPLS-DA score plots of the salivary metabolome profiles of AS patients and HCs detected by GC-MS analysis. The *x* axis and *y* axis indicate the first and second principal components, respectively. (E) S-plot from the OPLS-DA model of the salivary metabolome of AS patients and HCs. Two metabolites with increased or decreased levels were selected from this S-plot. (F) Heatmap of significantly different salivary metabolites between AS patients and HCs; *, *P < *0.05; **, *P* < 0.01; ***, *P* < 0.001; #, q < 0.05; ##, q < 0.01; ###, q < 0.001.

Among the 48 examined cytokines, the levels of two cytokines of the IL-6 family, sIL-6Rα and IL-11, were increased in AS saliva (Fig. 2A). In addition, the levels of five cytokines of the IL-10 family, including IL-20, IL-26, IL-28A, IL-29, and IL-10, were increased in AS saliva. Furthermore, three IL-12-related cytokines, IL-12p70, IL-12p40, and IL-27, were enriched in AS saliva. The levels of two interferons, IFN-α2 and IFN-β, were also increased in AS saliva (Fig. 2A and B). Finally, IL-2, osteocalcin, thymic stromal lymphopoietin (TSLP), and MMP-3 levels were also enriched in AS saliva (Fig. 2A).

Next, we examined the alterations of these 48 cytokines in AS serum and compared them to AS saliva. We observed that levels of IFN-α2, IL-12p70, and IL-29 were enriched in AS saliva but reduced in AS serum when compared separately to the levels observed in HC saliva or serum (Fig. 2B). Thirteen cytokines (IL-11, MMP-3, IFN-β, IL-2, sIL-6Rα, IL-10, IL-12p40, IL-20, IL-26, IL-27, IL-28A, osteocalcin, and TSLP) were enriched in only AS saliva but had no differences in levels between AS and HC serum (Fig. 2A). In contrast, IL-6, IL-23, soluble cluster of differentiation 163 (sCD163), chitinase 3-like 1, and soluble tumor necrosis factor receptor 1 (sTNFR1) were enriched and IL-19, IL-35, TNF superfamily member 14 (TNFSF14), and MMP-2 were depleted in AS serum but not AS saliva (Fig. 2C). These results suggest that alterations in salivary cytokines are likely derived from the oral cavity.

### Multiple metabolites are enriched in the saliva of AS patients compared to HCs.

To explore the alterations in the AS salivary metabolome, we assayed salivary metabolites in both AS and HC subjects using gas chromatography-mass spectrometry (GC-MS). A total of 56 metabolites, including 18 amino acids, 15 fatty acids, 5 amines, 4 aromatic acids, 3 monosaccharides, and 3 alcohols, were identified. 5-Aminovaleic acid had the highest peak area of all the identified metabolites in both AS and HC saliva. Furthermore, it was the only metabolite detected in all AS and HC subjects. Salivary metabolites whose mean peak areas also ranked in the top 10 in both groups were glycine, d-rhamnose, d-galactose, putrescine, and lactic acid, although they did not rank in the same order in AS and HC individuals.

To visualize the differences in salivary metabolome profiles between AS patients and HCs, we performed prthogonal projections to latent structures discriminant analysis (OPLS-DA), which is a supervised multivariate approach (Fig. 2D). OPLS-DA score plots validated by permutation analysis indicated that AS patients were clearly separated from HCs (R^2^X = 0.401; R^2^Y = 0.818; Q^2^ = 0.279). Seven metabolites, namely, *N*-acetyl-d-glucosamine, l-threonine, putrescine, phosphorylethanolamine, maleic acid, cadaverine, and 2-hydroxyisocaproic acid, had values of variable importance for projection in the OPLS-DA analysis greater than the threshold value of 1.5, indicating that these substances are of great significance for model construction. To identify candidate biomarkers, we performed an S-plot analysis. As shown in Fig. 2E, *N*-acetyl-d-glucosamine and phosphorylethanolamine were dispersed at both ends and are potential biomarkers.

We observed that the peak areas of 26 salivary compounds detected in the GC-MS analysis were significantly different between AS patients and HCs (Fig. 2F). On one hand, 25 metabolites were significantly enriched in AS saliva. Of the 12 AS-enriched amino acids, three were essential amino acids that cannot be synthesized by humans (l-threonine, l-lysine, and l-valine), seven were nonessential amino acids (l-proline, l-alanine, l-serine, l-ornithine, glycine, l-tyrosine, and 2-aminomalonic acid), and two were nonproteinogenic amino acids (pipecolic acid and 5-aminovaleric acid). The five AS-enriched fatty acids were malic acid, fumaric acid, 3-hydroxybutyric acid, glutamic acid, and pentanedioic acid. In addition, AS-enriched salivary metabolites also included two monosaccharides (d-rhamnose and d-galactose), two amines (cadaverine and putrescine), *N*-acetyl-d-glucosamine, benzenepropanoic acid, l-5-oxoproline, and 4-hydroxybenzeneacetic acid. In contrast, phosphorylethanolamine was detected in only HC saliva (Fig. 2F).

### Salivary bacteria, cytokines, and metabolites as potential biomarkers for the diagnosis of AS.

**FIG 3:**
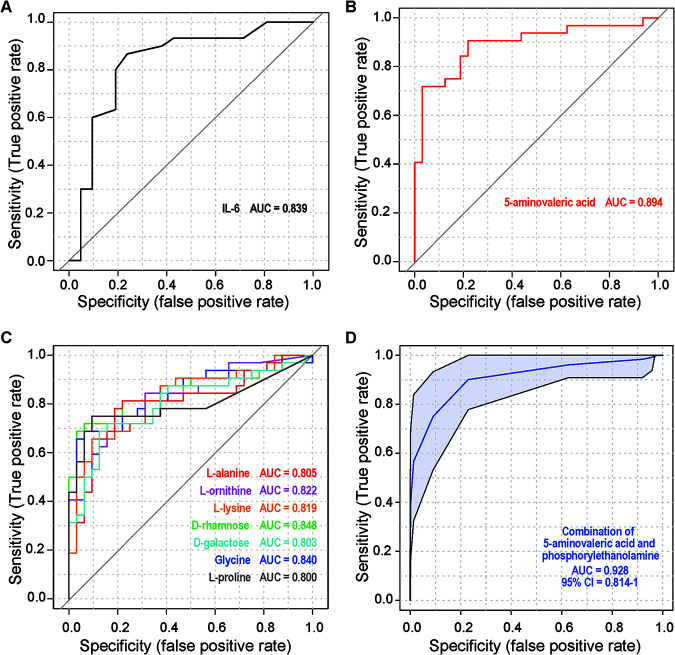
Diagnostic performance of serum or salivary markers for the discrimination of AS patients from HCs. ROC curve analysis to evaluate the discriminatory potential of serum biomarkers (A), salivary biomarkers (B and C), or their combination (D). Only markers with an AUC > 0.8 are shown.

The receiver operating characteristic (ROC) curve method was used to explore potential diagnostic biomarkers of AS from serum cytokines and salivary bacteria, cytokines, or metabolites. With an area under the curve (AUC) threshold of >0.8, 9 potential biomarkers were observed; these included one serum cytokine (IL-6, AUC = 0.84) (Fig. 3A) and eight salivary metabolites: 5-aminovaleric acid (AUC = 0.89; Fig. 3B), d-rhamnose (AUC = 0.85), glycine (AUC = 0.84), l-ornithine (AUC = 0.82), l-lysine (AUC = 0.82), l-alanine (AUC = 0.81), d-galactose (AUC = 0.80), and l-proline (AUC = 0.80; Fig. 3C). Among these metabolites, the combination of 5-aminovaleric acid and phosphorylethanolamine produced an AUC of 0.928 (Fig. 3D).

### Correlations between AS-altered salivary metabolites, microbes, cytokines, and disease indicators.

To evaluate the potential links between the AS-altered salivary microbiota, cytokines, metabolites, and disease indicators, we performed a Spearman correlation analysis. Correlation coefficients (in absolute value) of greater than 0.4 and *P *values of <0.01 were used as significance thresholds.

**FIG 4:**
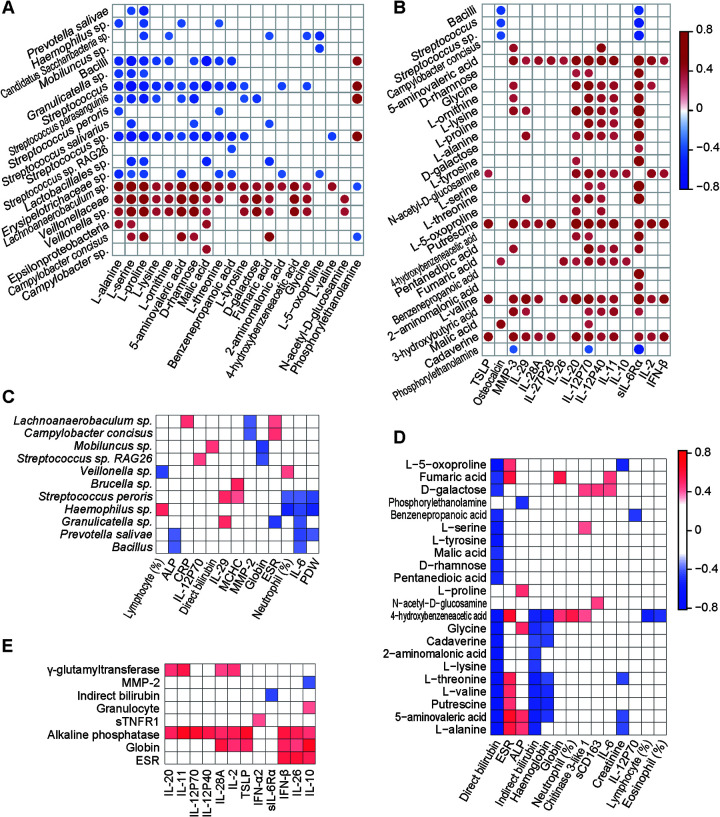
Networks between bacteria, cytokines, and metabolites in saliva and their potential links with systemic immunity and disease activity. (A) Correlations of salivary bacteria with salivary metabolites. (B) Correlations of salivary bacteria and metabolites with salivary cytokines. (C) Correlations of salivary bacteria with hematological variables and serum cytokines. (D) Correlations of salivary metabolites with hematological variables and serum cytokines. (E) Correlations of salivary cytokines with hematological variables and serum cytokines.

*Firmicutes* taxa contributed to nearly 88% of the correlations between AS-altered salivary bacterial taxa and metabolites (Fig. 4A). Specific AS-enriched metabolites, such as l-proline, d-rhamnose, malic acid, and 5-aminovaleric acid, were negatively correlated with certain AS-depleted microbes, such as Streptococcus parasanguinis and Streptococcus sp., but positively correlated with specific AS-enriched microbes, such as Campylobacter concisus, Campylobacter sp., *Veillonella* sp., and *Lachnoanaerobaculum* sp. In addition, AS-depleted phosphorylethanolamine was positively correlated with AS-depleted microbes such as Streptococcus parasanguinis and Streptococcus sp.

Twenty AS-altered metabolites were significantly correlated with one or more of ten AS-altered cytokines in the saliva (Fig. 4B). 5-Aminovaleric acid, l-alanine, d-rhamnose, and putrescine were positively correlated with at least five cytokines (sIL-6Rα, IL-12p70, IL-20, IL-29, and MMP-3), indicating their close links with oral immunity. l-Ornithine, l-proline, l-serine, l-threonine, l-tyrosine, malic acid, and pentanedioic acid were positively correlated with two or three cytokines, such as *N*-acetyl-d-glucosamine with IL-12p40, IL-12p70, and sIL-6Rα, or pipecolic acid with IL-20, IL-10, or IFN-β, showing their moderate links with oral immunity. In addition, 2-aminomalonic acid, 4-hydroxybenzeneacetic acid, and l-lysine were positively correlated with only sIL-6Rα, but phosphorylethanolamine was negatively correlated with sIL-6Rα. Interestingly, sIL-6Rα was correlated with 83% of the listed metabolites, indicating its important role in the cross talk between salivary metabolites and host immunity.

Only eight correlations were observed between AS-altered salivary bacteria and salivary inflammatory cytokines. Bacilli, Streptococcus, and Streptococcus sp. were negatively correlated with osteocalcin and sIL-6Rα. Campylobacter concisus was positively associated with IL-12p40 and MMP-3 (Fig. 4B).

AS-altered salivary bacteria were intensively correlated with blood immune biomarkers (Fig. 4C). AS-depleted salivary members of the Bacilli group (*Bacillus*, *Granulicatella* sp., Streptococcus peroris, and Streptococcus sp. RAG26), along with Prevotella salivae from *Bacteroidetes*, were negatively correlated with at least one AS-increased serum factor, including IL-6, ESR, alkaline phosphatase (ALP), globulin, or neutrophils, but positively correlated with at least one of the AS-depleted factors (IL-12p70 and IL-29). AS-enriched salivary *Veillonella* sp. were positively correlated with the neutrophil percentage but negatively correlated with the lymphocyte percentage, indicating their potential regulatory effects on leukocytes (Fig. 4C). More importantly, AS-enriched salivary Campylobacter concisus and *Lachnoanaerobaculum* sp. were positively correlated with ESR and/or C-reactive proteins (CRP) but negatively correlated with MMP-2, indicating their potential important roles in AS development.

AS-altered salivary metabolites were mainly correlated with erythrocyte-related bioprocesses (Fig. 4D). Bilirubin is the breakdown product of heme from the senescent hemoglobin of erythrocytes, and almost all AS-enriched salivary metabolites were negatively correlated with direct bilirubin. Among these metabolites, specific amino acids and their products, such as 5-aminovaleric acid, l-valine, and putrescine, were also negatively correlated with indirect bilirubin or hemoglobin and positively correlated with ESR and ALP, suggesting their close link with erythrocyte-related bioprocesses. In addition, l-serine, d-galactose, fumaric acid, *N*-acetyl-d-glucosamine, and 4-hydroxybenzeneacetic acid were positively correlated with at least one of the following factors: globin, chitinase 3-like 1, sCD163, and IL-6. In contrast, 4-hydroxybenzeneacetic acid was negatively correlated with the lymphocyte percentage and eosinophil percentage, and benzenepropanoic acid was correlated with IL-12p70, suggesting that these metabolites may be associated with immunity.

AS-altered salivary cytokines were strongly positively correlated with specific indicators of liver function (Fig. 4E). These positive correlations included AS-enriched salivary IL-2, IL-10, IL-11, IL-12p40, IL-12p70, IL-20, IL-26, IL-28A, TSLP, and IFN-β with serum ALP; salivary IL-2, IL-28A, IL-11 and IL-20 with serum gamma-glutamyl transpeptidase (GGT); and salivary IL-26, IFN-β, IL-10, IL-2, IL-28A, and TSLP with globin. All of the serum ALP, GGT, and globin levels are important indicators of liver function. Moreover, positive correlations were also detected between salivary IL-26, IFN-β, or IL-10 and ESR, between salivary IL-10 and blood granulocytes, and between salivary IFN-α2 and serum sTNFR1.

### Microbes and supernatants of AS saliva induce more cytokine production by THP-1-derived macrophages than those of HC saliva.

**FIG 5:**
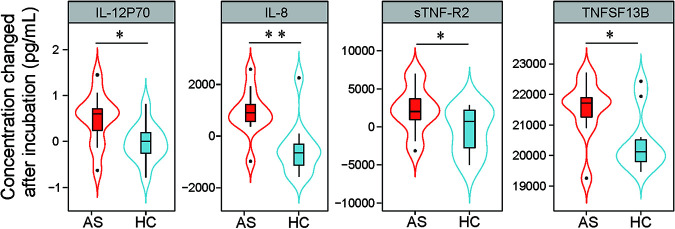
Salivary microbiota or supernatants of AS patients induced the production of higher levels of cytokines from THP-1-derived macrophages than did those of HCs; *, *P < *0.05; **, *P < *0.01; ***, *P < *0.001.

To further explore the potential impact of the AS-altered salivary microbiota on immunity, we compared the ability of AS salivary microbes and supernatants to induce cytokine production with that of HC salivary microbes and supernatants *in vitro*. We initially assessed their effects on human oral OKF6 keratinocytes. To prevent salivary microbes from changing their composition during coculture with cells, we inactivated them at 80°C for 15 min and confirmed that they could not reproduce. After 48 h of coculture of OKF6 keratinocytes with salivary microbes or supernatants, there was no significant difference in cytokine levels in the culture medium with added salivary microbes or supernatants between AS and HC individuals. In addition, specific oral small molecules can penetrate the mucosa, and some oral microbes may break through the oral mucosa because teeth pass through the mucosa, leading to a lack of integrity in the tight junctions. Macrophages provide a first line of defense if this happens. After incubation with cell line THP-1-derived macrophages for 48 h, heat-inactivated AS salivary microbes caused a greater increase in TNF13B in the culture medium than did those from HCs, whereas AS salivary supernatant caused a greater increase in sTNFR2, IL-12p70, and IL-8 than HC salivary supernatant, indicating the important potential role that AS saliva plays in the stimulation of inflammation (Fig. 5).

### Tooth brushing decreases the levels of inflammatory cytokines and salivary metabolites in AS saliva compared with HC saliva.

**FIG 6:**
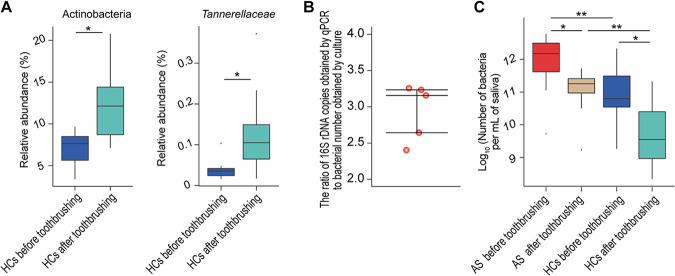
Decreased bacterial abundance was the primary change in the salivary microbiota of both AS patients and HCs after tooth brushing. (A) The relative abundances of two bacterial taxa were altered in HCs from before to after tooth brushing. (B) Five replicates of the ratio of 16S rRNA genes copies of Escherichia coli ATCC 25922 obtained by qPCR to the number of ATCC 25922 obtained by plate counting using the same volume of fresh fermentation broth of ATCC 25922. (C) The number of bacteria in the saliva of AS patients and HCs before and after tooth brushing. The 16S rRNA gene copies in the saliva obtained by qPCR were converted to bacterial numbers based on the ratio of 16S rRNA genes copies of Escherichia coli ATCC 25922 obtained by qPCR to ATCC 25922 number obtained by culture; *, *P < *0.05; **, *P < *0.01; ***, *P < *0.001.

The 16S rRNA gene sequencing analysis showed there were no significant alterations in the relative abundances of salivary bacterial taxa before and after tooth brushing in AS patients. In HC saliva, only the relative abundance of the phylum *Actinobacteria* and the family *Tannerellaceae*, which belongs to the phylum *Bacteroidetes*, were depleted after tooth brushing, while the relative abundance of other bacterial taxa remained unchanged (Fig. 6A). Importantly, the quantitative PCR (qPCR) results showed that after taking the extraction losses into account, the number of salivary bacteria was significantly higher in AS patients than in HCs both before and after brushing (Fig. 6B and C). Furthermore, after tooth brushing, the number of salivary bacteria decreased nearly 90% in both the AS patients and HCs (Fig. 6C). Therefore, the salivary microbiota alterations detected after tooth brushing in both AS patients and HCs were primarily observed as decreases in the total abundance.

**FIG 7:**
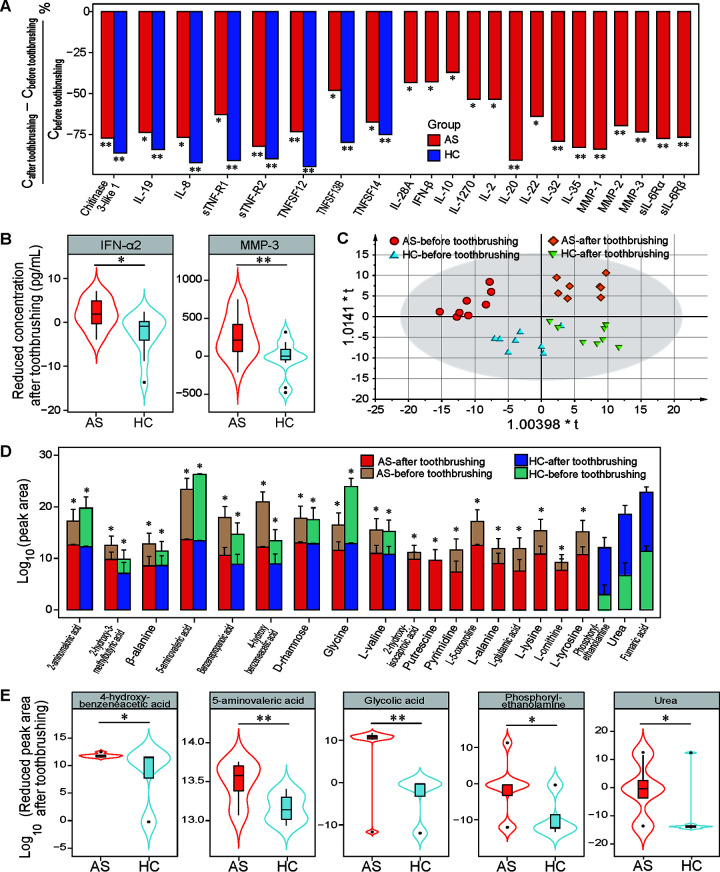
The levels of harmful salivary metabolites and cytokines were reduced to a greater extent in AS patients than HCs after tooth brushing. (A) Percentage of decreased cytokines after tooth brushing in AS patients and/or HCs. (B) Cytokines with significant differences in concentration changes between AS patients and HCs after tooth brushing. (C) OPLS-DA score plots of the salivary metabolome profiles of AS patients and HCs before and after tooth brushing. (D) Metabolites with significant changes in peak area before and after tooth brushing in AS patients and/or HCs. (E) Metabolites with significant differences in altered peak areas between AS patients and HCs after tooth brushing; *, *P < *0.05; **, *P < *0.01; ***, *P < *0.001.

To further explore the potential effect of salivary microbes and their products on oral immunity, we compared the alterations in salivary cytokines in AS patients and HCs before and after tooth brushing. Compared to those observed before tooth brushing, the levels of seven cytokines, including TNFSF13B, chitinase 3-like 1, GP130 (sIL-6Rβ), IL-8, IL-19, sTNFR1, and sTNFR2, were decreased in the saliva of both AS patients and HCs after tooth brushing. However, the expression levels of 16 cytokines, including IL-2, sIL-6Rα, IL-10, IL-12p40, IL-12p70, IL-20, IL-22, IL-32, IL-35, TNFSF12, TNFSF14, MMP-1, MMP-2, MMP-3, osteopontin, and TSLP, were decreased in AS but not HC saliva after tooth brushing (Fig. 7A). Next, we compared the altered levels of cytokines between HC and AS patients during this process and found greater decreases in IFN-α2 and MMP-3 levels in AS saliva than in HC saliva (Fig. 7B).

The OPLS-DA results showed that the metabolome profiles before and after tooth brushing were separate in AS patients (Fig. 7C). More importantly, the metabolome profile of AS patients was clustered separately from that of HCs before tooth brushing, but the profile cluster distances decreased and were almost indistinguishable from each other after tooth brushing. The paired analysis showed that the levels of 2-aminomalonic acid, 2-hydroxy-3-methylbutyric acid, 5-aminovaleric acid, and β-alanine decreased significantly from before to after tooth brushing in both groups (Fig. 7D). However, the levels of 2-hydroxyisocaproic acid, 4-hydroxybenzeneacetic acid, d-rhamnose, glycine, l-glutamic acid, benzenepropanoic acid, l-lysine, and l-valine only decreased in AS patients. Interestingly, fumaric acid and urea levels increased in HCs after tooth brushing (Fig. 7D). Moreover, the reductions in 5-aminovaleric acid, 4-hydroxybenzeneacetic acid, and glycolic acid between before and after tooth brushing were greater in AS patients than HCs, but the increased levels of phosphorylethanolamine and urea were greater in HCs than AS patients during this process (Fig. 7E).

## DISCUSSION

The role of microbes in diseases such as AS has attracted extensive attention (7, 21–24). The oral microbiota is the second-largest microbial community of the human body and can affect multiple systems, such as the digestive system, respiratory system, and immune system. In this work, we performed an integrated analysis of the microbiota, cytokines, and metabolomes in AS saliva and explored their potential effects on AS *in vivo* and *in vitro*. Our results showed that, compared to that of HCs, the saliva of AS patients was depleted of Bacilli members such as Streptococcus, enriched with Clostridia members such as *Veillonellaceae*, and enriched with opportunistic pathogens from *Proteobacteria*, such as Brucella sp. and Campylobacter concisus. Furthermore, 16 salivary cytokines, such as IL-12p40 and IL-12p70, and 26 metabolites, such as 5-aminovaleric acid, cadaverine, and putrescine, were significantly enriched in AS patients versus HCs. After tooth brushing, a greater number of cytokines and metabolites were decreased in AS saliva than in HC saliva. Microbes and supernatants of AS saliva induce the production of more cytokines, such as IL-12p70 and IL-8, by THP-1 macrophages than do those of HC saliva. These results indicate that there are harmful alterations in the salivary microbiota of AS patients.

The alterations in the AS salivary microbiota exhibited unique characteristics. For instance, some AS-altered bacterial taxa are different from those known in oral diseases. For example, compared to that observed in HC saliva, the relative abundances of Streptococcus mitis, Streptococcus parasanguinis, and *Prevotella* sp. are increased and the relative abundances of Campylobacter concisus and *Veillonella* sp. are reduced in the saliva of patients with chronic periodontitis, whereas in AS saliva, the relative abundances of these bacteria are altered in the opposite direction (25). Bifidobacterium dentium, Lactobacillus salivarius, and Streptococcus sp. ot068 are significantly enriched in the saliva of caries patients; however, in AS patients, the relative abundance of the former two did not change significantly, whereas the relative abundance of Streptococcus and its members was decreased (26). In addition, the alterations in the AS salivary microbiota are different from those known in patients with several other autoimmune diseases. For example, compared to that observed in HCs, the relative abundance of *Firmicutes* and Streptococcus was increased and the relative abundance of *Bacteroidetes* was decreased in the saliva of patients with autoimmune polyendocrinopathy syndrome type 1. However, in AS saliva, the relative abundances of *Firmicutes* and *Bacteroidetes* were not significantly altered, whereas the relative abundance of Streptococcus was decreased (27). *Veillonella* was enriched in the saliva of both AS and rheumatoid arthritis (RA) patients, but enrichment of Lactobacillus salivarius, which is highly correlated with RA, was not observed in AS patients (13). Moreover, AS-depleted salivary Streptococcus is enriched in the saliva of primary Sjogren's syndrome patients with normal salivation (14). AS-enriched salivary Campylobacter concisus is depleted in the saliva of patients with Behcet’s disease (15). These above-mentioned characteristic alterations of the AS salivary microbiota indicate the unique interactions between the host and the salivary microbiota in AS patients and the great potential of the salivary microbiota for use in diagnosis, although the comparison of different studies will be limited by participants and methods.

In the present study, opportunistic pathogens were enriched in the saliva of AS patients. For example, Brucella sp., which is a member of the brucellosis-causing genus Brucella, was enriched in AS patient saliva. Currently, approximately 500,000 cases of human brucellosis are reported worldwide annually (28), among which osteoarticular brucellosis is the most common. Moreover, sacroiliitis, spondylitis, and peripheral arthritis are the three most common forms of osteoarticular involvement (29). Correspondingly, our results showed that Brucella sp. was present in the saliva of 87.10% of AS patients but only 36.30% of HCs. Likewise, Campylobacter members primarily colonize the human oral cavity and cause millions of illnesses every year. Therefore, their enrichment may be an important factor in the onset or deterioration of AS. Among Campylobacter species, AS-enriched Campylobacter concisus has been linked to prolonged diarrhea, gastroesophageal reflux disease, Barrett’s esophagus, and inflammatory bowel disease (30). Interestingly, an HLA-B*27:05 ligand that is dependent on endoplasmic reticulum-resident aminopeptidase was observed to be highly similar to the sequence of a protein from Campylobacter jejuni, indicating the potential of Campylobacter jejuni as an arthritogenic bacteria (31). In addition, enrichment of *Veillonella* was also previously observed in the gut of AS patients (32), as well as in the saliva. *Veillonella* are typically regarded as contaminant bacteria but may lead to serious infections such as bacteremia, endocarditis, meningitis, peritonitis and osteomyelitis (33). Furthermore, Veillonella parvula was reported to be involved in bone and joint infections (34).

Our results showed that cytokines were significantly enriched in the saliva of AS patients. Interestingly, some cytokines enriched in AS saliva play an important role in promoting the proliferation and differentiation of immune cells. IL-2 can promote lymphocyte growth, proliferation, and differentiation (35); IL-11 can stimulate the development of mature cells such as plasma cells and B cells (36); IL-20 can promote the proliferation and differentiation of keratinocytes in inflammation (37); and IL-26 is associated with Th17 cells (37). In addition, IL-12p40, which can promote the differentiation of Th1 cells from Th0 cells and can induce the cytotoxic activity of natural killer cells, is an important component of IL-12p70 and has some of the proinflammatory activity of IL-12p70 (38). The IL-12p70-induced differentiation of Th1 cells may be associated with the development of organ-specific autoimmune diseases. Furthermore, IL-12p40-deficient mice are resistant to experimental autoimmune encephalomyelitis, whereas IL-12p35^−/−^ mice are susceptible, indicating that IL-12p40 loss may be more important than IL-12 loss for the development of autoimmune inflammation (39). In addition, some cytokines enriched in AS saliva are closely related to the inflammatory response. sIL-6Rα can bind to IL-6 to stimulate the reverse inflammatory pathway (36). Correspondingly, the inhibition of IL-6 transsignaling, wherein the complex of IL-6 and sIL-6R can stimulate cells that do not express IL-6R (such as endothelial cells and smooth muscle cells), is sufficient to block disease in mouse models of inflammatory bowel disease and RA (40). IL-10 is primarily an anti-inflammatory cytokine (37); IL-27 is a negative regulator of IL-12 (38); TSLP is a “master switch” for allergic inflammation (41); and MMP-3 can degrade extracellular matrix proteins and aggravate the inflammatory response (42). Importantly, increased MMP-3 levels have also been observed in supraspinous ligament samples from AS patients, whereas the degradation of extracellular matrix components is a pathological feature of chronic arthritis (43). Some cytokines enriched in AS saliva are associated with infections, such as type I interferons (IFN-α2 and IFN-β) upon bacterial infection, and type III interferons (IL-28A and IL-29) upon both viral and bacterial infections (37). Furthermore, osteocalcin, the levels of which are enriched in AS saliva, is crucial for the maintenance of bone mineralization (44). These results indicate that oral inflammation exists in AS patients.

Our results showed that the saliva of AS patients was enriched in several potentially harmful metabolites. For example, AS-enriched 2-aminomalonic acid is associated with genotoxicity. AS-enriched pentanedioic acid can induce oxidative stress, excitotoxicity, and mitochondrial dysfunction in the rat brain (45). AS-enriched 5-oxoproline, which is a product of disordered glutathione metabolism, may lead to metabolic acidosis (46). AS-enriched 5-aminovaleric acid, which is an analog of γ-aminobutyric acid (GABA), has been reported to inhibit GABA uptake and the activity of GABA aminotransferase. Both AS-enriched putrescine and cadaverine are produced mainly through the decarboxylation of arginine or lysine by plants, animals, and microorganisms. Putrescine plays an important role in cellular metabolism, contributing to bacterial cell-cell signaling, cell division, and motility and is essential for the virulence of Salmonella, *Legionella*, *Francisella*, *Shigella*, and avian pathogenic Escherichia coli (47). Moreover, putrescine or cadaverine at concentrations as high as those in specific foods produce cytotoxic effects in HT29 intestinal cell cultures by inducing necrosis (48). Correspondingly, our results showed that after tooth brushing, the levels of putrescine and 5-oxoproline were decreased in only AS patients. These results suggest the potential hazards of AS salivary metabolites to health.

Our *in vitro* and *in vivo* results showed that salivary components of AS patients, including microbiota and metabolites, may have higher proinflammatory effects. The salivary levels of IL-8, IL-19, TNFSF13B, TNFSF12, sTNFR1, sTNFR2, GP130, and chitinase 3-like 1 before tooth brushing were not significantly different between AS patients and HCs. After tooth brushing, these cytokines were significantly reduced in both HCs and AS patients, accompanied by a reduction in salivary microbes and metabolites, indicating that these cytokines can be affected by salivary components, especially those present in both AS patients and HCs. Further results showed that salivary microbes or supernatants of AS patients induced the production of higher levels of TNFSF13B or IL-8 and STNFR2 by THP-1-derived macrophages than those of HCs, suggesting that AS saliva has increased virulence. On the other hand, the salivary levels of sIL-6Rα, IL-2, IL-10, IL-12p70, IL-20, IL-28A, MMP-3, and IFN-β before brushing were significantly higher in AS patients than in HCs. After tooth brushing, their levels decreased in AS patients but not HCs. This finding indicates that these cytokines can be affected by salivary components, especially microbes or metabolites that are different between AS patients and HCs. Two lines of evidence further verified this point: (i) the extent of MMP-3 reduction before and after brushing was significantly greater in AS saliva than in HC saliva, and (ii) the supernatants of AS saliva stimulated THP-1-derived macrophages to produce increased amounts of IL-12p70. In addition, our results showed that most AS-enriched microbes and metabolites were positively correlated with inflammatory cytokines in saliva. Therefore, these results indicate that the altered salivary microbiota and metabolites may play a role in the development of AS.

There are some limitations to this study, which include the small number of AS patients evaluated due to strict exclusion criteria, the limited investigation of the influence of diet and/or the living or working environment, and the impacts of potential biases, including the uneven distribution of education, heredity, and customs among groups. The importance of the data provided in this study and their potential contributions to understanding pathogenesis and treatment merits further investigation in well-designed, longitudinal, large, confirmatory studies.

In summary, AS patients have an enrichment of salivary opportunistic pathogens, inflammatory cytokines, and toxic metabolites, which are closely associated with the severity of AS. The AS-altered salivary microbiota is an important reason for the increased levels of salivary cytokines and metabolites. Oral health care, especially of the microbiota, should receive greater attention in the daily health care of AS patients.

## MATERIALS AND METHODS

### Participants and samples.

Only patients who met both the 1984 modified New York classification criteria of AS (49) and all of the following criteria were enrolled in this study: no oral diseases detected during an oral examination; no other serious diseases or health problems, such as obesity, diabetes, tumors, or gastrointestinal disorders, viral hepatitis, fatty liver disease, alcoholic/drug-induced liver disease, cardiorenal dysfunction, circulatory system diseases, or nervous system diseases; and no antibiotics or probiotic drugs taken within the prior 3 months. A dietary questionnaire was used to exclude individuals who had specific dietary habits, such as a completely vegetable-based diet. Both the AS patients and HCs evaluated in the present study maintained a similar daily diet (a Southeast China dietary habit of rice as the staple food and bland dish styles) and geographic location. All participants signed informed consent forms, and the study protocol was approved by the ethics committee of Zhejiang University (approval no. 2018-843).

All samples were collected from seven to nine o’clock in the morning. The first saliva sampling was conducted before any of the participants had breakfast, brushed their teeth, drank water, or cleaned their mouths. For saliva sampling, saliva was first collected in the mouth for at least 1 min and then drooled into a labeled 50-ml collection tube. This process could be repeated multiple times to collect at least 5 ml of saliva. Then, all participants brushed their teeth according to the Bass method (50), cleaned their tongue, rinsed thoroughly until the spit water was clear, and slowly drank 200 ml of water. One hour later, each participant drank 100 ml of water and, after another 10 min, saliva sampling was carried out as described above. Blood samples were collected before tooth brushing, some of which were immediately used for biochemical tests, and the remaining samples were separated into serum and blood cells. All samples were stored at –80°C until use.

### Assessment of hematological variables.

The erythrocyte sedimentation rate (ESR) was measured with a Vacuette SRS100/II instrument (Greiner Bio-One, Frickenhausen Germany). C-reactive protein (CRP) levels were tested using a HITACHI LABOSPECT 008 (Hitachi, Tokyo, Japan). Hematological variables such as the percentage and counts of neutrophils, lymphocytes, monocytes, eosinophils and basophils were tested using a Sysmex XN-2000 instrument (Sysmex, Japan). Measures of liver function, including total protein, albumin, globulin, alkaline phosphatase (ALP), direct bilirubin, indirect bilirubin, and gamma-glutamyl transpeptidase (GGT) levels, were assessed by standard methods using a 7600-210 automatic analyzer (Tokyo, Japan). Renal functions, including serum urea nitrogen, creatinine, and uric acid, were determined using a Cobas 8000 system (Roche Diagnostics, Mannheim, Germany).

### Analysis of AS disease activity.

Patients were monitored with the following assessments: patient global assessment score, physician global assessment of disease activity, and the six individual questions of the Bath ankylosing spondylitis activity disease activity index (BASDAI), including fatigue, spinal pain, pain and swelling of peripheral joints, pain at entheseal sites, severity of morning stiffness, and duration of morning stiffness (with 10 representing a duration of 2 h or longer). All scores were recorded on a visual analog scale ranging from 0 (representing the normal situation) to 10 cm (representing the most extreme situation), where BASDAI = 0.2 × (fatigue + spinal pain + pain and swelling of peripheral joints + pain at entheseal sites + 0.5 × [severity of morning stiffness + duration of morning stiffness]). In addition, the AS disease activity score (ASDAS) was calculated using the following formula: ASDAS = 0.12 × back pain + 0.06 × duration of morning stiffness + 0.11 × patient global + 0.07 × peripheral pain/swelling + 0.58 × ln(CRP + 1) (51).

### DNA extraction, sequencing, and data processing.

Genomic DNA was manually extracted from the centrifuged sediment of 500 μl of saliva according to a previously reported method (52). The V3-V4 region of the 16S rRNA gene was amplified using the primer pair 338F (5′-ACTCCTACGGGAGGCAGCAG-3′) and 806R (5′-GGACTACHVGGGTWTCTAAT-3′), the 5′ ends of which were tagged with sample-specific barcodes. The PCR conditions were as follows: initial denaturation at 98°C for 30 s; 35 cycles of 98°C for 10 s, 54°C for 30 s, 72°C for 45 s; and a final extension at 72°C for 10 min. Following purification and quantitation steps, the amplicons were sequenced using the Illumina MiSeq platform (Illumina Inc., San Diego, CA) (53). Paired-end reads were merged by FLASH and then filtered according to fqtrim (v0.94) to obtain high-quality clean tags. Chimeric sequences were filtered using Vsearch (v2.3.4). Sequences with ≥97% similarity were assigned to the same operational taxonomic units (OTUs) using Vsearch (v2.3.4). Representative sequences were chosen for each OTU, and taxonomic data were then assigned to each representative sequence using the RDP (Ribosomal Database Project) classifier. The differences in the dominant species in different groups and multiple sequence alignments were analyzed using MAFFT (v7.310) to study the phylogenetic relationships of different OTUs. The alpha and beta diversity were calculated using QIIME (v1.8.0).

### Metabolomic profiling and analysis.

To prepare samples for gas chromatography-mass spectrometry (GC-MS)-based metabolite profiling, each saliva sample underwent the following steps. For each 200 μl of saliva supernatant acquired by centrifugation at 10,000 × *g* for 3 min, 800 μl of methanol was added. Following thorough mixing and centrifugation at 18,000 × *g* for 15 min, the supernatant was filtered through a 0.22-μm membrane filter. Then, 20 μl of heptadecanoic acid (1 mg/ml) was added as an internal reference, and each filtered supernatant was dried at room temperature under nitrogen. Subsequently, 50 μl of methoxypyridine solution (15 mg/ml) was added to the dried mixture. The new solution was then mixed for 1 min, sealed in an airtight container, and kept at 37°C for 24 h. Then, 50 μl of N,O-bis(trimethylsilyl)acetamide containing 1% trimethylchlorosilane was added, and the solution was mixed well and kept at 70°C for 2 h for derivatization.

The pretreated samples were analyzed by an Agilent 7890A/5975C GC-MS (Santa Clara, CA, USA). Both the integration of raw data and extraction of mass spectra were conducted using Agilent MassHunter Qualitative Analysis Version B.07.00. The peaks of the mass spectra were compared to those in the NIST17 database. The peak areas of compounds with a match score of ≥80 were quantified according to the internal standard. Orthogonal projections to latent structures discriminant analysis (OPLS-DA) and S-plot analyses were performed using SIMCA-14.1.

### Analysis of cytokines.

In saliva or serum, the concentrations of 48 cytokines, namely, interleukin 1β (IL-1β), IL-2, IL-4, IL-6, IL-8, IL-10, IL-11, IL-12p40, IL-12p70, IL-17A, IL-17F, IL-19, IL-20, IL-21, IL-22, IL-23, IL-25, IL-26, IL-27p28, IL-28A, IL-29, IL-31, IL-32, IL-33, IL-34, IL-35, interferon α2 (IFN-α2), IFN-γ, IFN-β, matrix metalloproteinase 1 (MMP-1), MMP-2, MMP-3, soluble cluster of differentiation 163 (sCD163), soluble CD40 ligand, soluble IL-6 receptor α (sIL-6Rα), sIL-6Rβ, tumor necrosis factor α (TNF-α), TNF superfamily member 8 (TNFSF8), TNFSF12, TNFSF13, TNFSF13B, TNFSF14, soluble TNF receptor 1 (sTNFR1), sTNFR2, osteocalcin, osteopontin, pentraxin 3, thymic stromal lymphopoietin (TSLP), and chitinase 3-like 1 were measured with commercially available enzyme-linked immunosorbent assay kits using the Bioplex 200 System by following the manufacturer’s recommendations (Bio-Rad, Hercules, CA).

### Plate counting.

The cryopreserved Escherichia coli strain ATCC 25922 was crossed onto a Luria-Bertani (LB; Oxoid, Thermo Fisher Scientific, China) plate and incubated at 37°C for 18 h. Then, a single clone was picked and cultured in 5 ml of LB liquid medium at 30°C with shaking at 200 rpm for 18 h. Next, 1 ml of the bacterial culture was used to generate 10^4^ to 10^9^ dilutions. Then, 100 μl of each dilution was coated onto an LB plate (three replicates for each dilution) and cultured for 18 h using the above-mentioned method, after which the number of colonies was enumerated.

### Quantitative PCR primers and thermocycling conditions.

The amount of total bacteria was estimated from the quantity of 16S rRNA gene copies as determined by quantitative PCR (qPCR) with the primers 338f (5′-ACTCCTACGGGAGGCAGCAG-3′) and 518r (5′-ATTACCGCGGCTGCTGG-3′). qPCR was performed with a ViiA7 real-time PCR system (Applied Biosystems, USA) using SYBR Premix Ex TaqTM (RR420A; TaKaRa, Tokyo, Japan). Each reaction mixture was prepared at a 10-μl final volume and contained 5 μl of TB green, 0.4 μl (1 μM) of each primer, 0.2 μl of ROX reference dye, 2 μl of DNA template, and 2 μl of PCR-grade water. The reactions were hot-started at 95°C for 30 s, followed by 40 cycles of 95°C for 5 s and 56°C for 34 s, and the subsequent melt curve stage of 95°C for 15 s, 60°C for 1 min, and 95°C for 15 s. A plasmid harboring the PCR-amplified 16S rRNA gene from E. coli ATCC 25922, which was similarly prepared as previously described (54), was used to produce a calibration curve of the 16S rRNA gene copy concentration versus the cycle quantification value. Calibration curves were generated using standards with 6 consecutive dilution steps (the number of bacteria ranged from 10^3^ to 10^9^ cells/ml). Sample DNA was analyzed in duplicate without dilution.

### Cell culture.

Both the telomerase-immortalized human oral epithelial OKF6/TERT2 cell line (OKF6) and the human monocytic THP-1 cell line were obtained from the Chinese Academy of Sciences (Shanghai, China). OKF6 cells were grown in keratinocyte medium (ScienCell, Carlsbad, CA, USA) supplemented with penicillin (100 U/ml) and streptomycin (100 μg/ml). Human THP-1 cells were maintained at a concentration between 2 × 10^5^ and 1 × 10^6^ cells/ml in RPMI 1640 containing 10% fetal bovine serum, HEPES (25 mM), 2-mercaptoethanol (0.05 mM), penicillin (100 U/ml), and streptomycin (100 μg/ml). All cells were incubated at 37°C in a humidified 5% (vol/vol) CO_2_ incubator.

### Coculture of saliva with cells.

To identify an appropriate condition for the thermal inactivation of salivary microbes, the precipitates of the centrifuged saliva were washed three times with phosphate-buffered saline (PBS, pH 7.4), resuspended in PBS, and incubated in a water bath at 70°C or 80°C for 15, 30, 60, or 120 min. Then, 50 μl of these suspensions was plated on brain heart infusion plates and sheep blood agar plates, which were cultured under anaerobic and aerobic conditions. Microbial growth was observed at 24, 48, and 72 h. A condition in which the inactivation time was short and the temperature was low was selected as the thermal inactivation condition for further research (55).

OKF6 or THP-1 cells were seeded at a density of 4 × 10^4^ cells per well in a 96-well plate and cultured overnight. THP-1 cells were differentiated into macrophages by treatment with 100 ng/ml phorbol 12-myristate 13-acetate (Sigma-Aldrich) and 0.3% bovine serum albumin in serum-free RPMI 1640 for 48 h. Then, macrophages derived from THP-1 cells were further cultured overnight in RPMI 1640 supplemented with 10% fetal bovine serum, penicillin (100 U/ml), and streptomycin (100 μg/ml). After the concentrations were determined using a Thoma cell-counting chamber, thermally inactivated salivary microbes were added to OKF6 cell cultures at a multiplicity of infection of 50, where the ratio of the number of microbes to the number of OKF6 cells was 50 (56). Salivary supernatants sterilized by filtration were separately added to other OKF6 cells or macrophages derived from THP-1 cells. Supernatants of these cocultures were sampled at 0, 12, 24, 36, and 48 h for cytokine assays.

### Statistical analysis.

The sample size was estimated with α = 0.05 and 1-β = 0.95 using PASS 15.0 (NCSS, UT, USA). For the results of the hematological variables, liver functions, renal functions, cytokines, metabolites, ESR, CRP, and α-diversity of the microbiota, the Kolmogorov-Smirnov test was used to check for normality, and the Mann-Whitney U test was used to compare any two data sets that were not normally distributed; otherwise, one-way ANOVA followed by the Student-Newman-Keuls method was used. The Wilcoxon rank-sum test combined with the Benjamini-Hochberg method was applied to compare bacterial taxa. Permutational multivariate analysis of variance (PERMANOVA) was performed using Adonis. Spearman’s rank correlation test was used to analyze the correlations between two variables. For all graphs, nonsignificant results (*P > *0.05 or q > 0.05) are left blank; *, *P < *0.05; **, *P < *0.01; ***, *P < *0.001; #, q < 0.05; ##, q < 0.01; and ###, q < 0.001.
